# An insight into intestinal mucosal microbiota disruption after stroke

**DOI:** 10.1038/s41598-017-18904-8

**Published:** 2018-01-12

**Authors:** Dragana Stanley, Robert J. Moore, Connie H. Y. Wong

**Affiliations:** 1 School of Health Medical and Applied Sciences, Central Queensland University, Bruce Highway, Rockhampton, Queensland 4702 Australia; 2 School of Science, RMIT University, Bundoora, Victoria 3083 Australia; 3 Infection and Immunity Program, Biomedicine Discovery Institute, Department of Microbiology, Monash University, Clayton, Victoria 3800 Australia; 4 Centre for Inflammatory Diseases, Department of Medicine, School of Clinical Sciences, Monash University, Clayton, Victoria 3168 Australia

## Abstract

Recent work from our laboratory has provided evidence that indicates selective bacterial translocation from the host gut microbiota to peripheral tissues (i.e. lung) plays a key role in the development of post-stroke infections. Despite this, it is currently unknown whether mucosal bacteria that live on and interact closely with the host intestinal epithelium contribute in regulating bacterial translocation after stroke. Here, we found that the microbial communities within the mucosa of gastrointestinal tract (GIT) were significantly different between sham-operated and post-stroke mice at 24 h following surgery. The differences in microbiota composition were substantial in all sections of the GIT and were significant, even at the phylum level. The main characteristics of the stroke-induced shift in mucosal microbiota composition were an increased abundance of *Akkermansia muciniphila* and an excessive abundance of clostridial species. Furthermore, we analysed the predicted functional potential of the altered mucosal microbiota induced by stroke using PICRUSt and revealed significant increases in functions associated with infectious diseases, membrane transport and xenobiotic degradation. Our findings revealed stroke induces far-reaching and robust changes to the intestinal mucosal microbiota. A better understanding of the precise molecular events leading up to stroke-induced mucosal microbiota changes may represent novel therapy targets to improve patient outcomes.

## Introduction

Stroke is highly prevalent and is one of the leading contributors to morbidity and mortality worldwide. Despite the debilitating neurological deficits, the major cause of death after stroke is bacterial pneumonia^1,2^. However, the precise mechanism behind the host immune functional impairment and its contribution to the weakened antimicrobial defence following stroke is still largely unknown. Furthermore, the origins of the resulting pneumonia, that is often a serious consequence of stroke, remain elusive. Often, no recognised pathogen is identified using common clinical microbiological methods, with little or no information obtained from blood and sputum samples^3^. Intriguingly, a number of large clinical studies have been unable to support the use of preventative antibiotics to limit post-stroke infections^4–7^. In fact, depletion of intestinal microbiota in mice using broad spectrum antibiotics prior to stroke induction resulted in increased post-stroke mortality unrelated to cerebral infarct size, and the survival rate was improved by recolonization of the post-stroke mice with complex gut microbiota^8^. Furthermore, a novel gut microbiota-brain axis interaction was recently revealed to explain how intestinal dysbiosis primes the immune system and alters host homeostasis to result in enhanced neuroinflammation after stroke^9^. These studies clearly highlight the important contribution of intestinal microbiota in post-stroke recovery outcome.

In addition, recent work from our laboratory has provided evidence that indicates selective bacterial translocation from the host gut microbiota to peripheral tissues (i.e. lung) plays a key role in the development of these post-stroke infections^10^, thus implicating gut epithelial mucosa and intestinal microbiota as the main players in post-stroke mortality. Importantly, the finding of differentially abundant phylotypes in the lung microbiota after stroke compared to gut microbiota implies that there is selectivity in barrier leakage, or survival, or expansion of bacteria that differs between sham-operated and post-stroke mice. In the present study, we propose that the intestinal mucosal microbiota is a battleground and a hotspot for bacterial translocation after stroke. Mucosal bacteria are located adjacent to the host epithelia and hence interact more closely with the host than the luminal bacteria. In fact, the mucosal bacteria represent part of the host’s first line of defence against opportunistic pathogens in the gut^11,12^. Moreover, mucosal bacteria have been demonstrated to interact with the host and influence host gene expression and wound healing processes^13,14^. Despite the awareness that the mucosal bacterial cohort is very distinct in both role and membership from the luminal and faecal microbiota, mucosa-associated bacteria are often overlooked. Therefore, in this study, we provide a detailed insight into the composition of the mucosal microbiota after stroke. Being closest to the host epithelium, we propose that the mucosal microbiota is the prime location to respond to stroke injury and potentially has the opportunity to either invade the host or launch epithelial healing responses to re-establish gut barrier integrity after stroke.

## Results and Discussion

In this study, we analysed and compared the microbial communities associated with mucosa across five gastrointestinal tract (GIT) sections: duodenum, jejunum, ileum, cecum, and colon, between the sham-operated (control) and post-stroke mice at 24 h. The tissue and samples obtained for this study came from the animals that underwent thorough neurological, immunological and cell based (flow cytometric) analysis, the findings of which has been published^10^. We demonstrated clear and consistent brain infarct size in all post-stroke animals (38.8 ± 3.6 mm^3^), compared to zero in sham-operated animals. Specifically, the animals examined for mucosal microbiota in the current study underwent detailed bacteriological analysis, whereby only mice from the post-stroke cohort showed evidence of bacterial translocation after surgery^10^. In addition, surgical stress, as represented by mice that underwent sham procedure, has been shown to affect microbial communities^15^. However, we did not find any difference in the incidence of infection between naïve or sham-operated mice^10^, indicating the potential shift in microbial communities in animals that underwent sham surgery compared to the naïve mice did not contribute to spontaneous infection, and as a result was not compared in this study. Therefore, we focused our current study to examine the intestinal mucosal microbial communities of sham-operated and post-stroke mice.

To explore the differences in microbial communities between the groups of mucosal samples, we used a redundancy analysis (RDA) as a method to summarise the variation in a set of response variables (ie. operational taxonomic units; OTUs) that can be explained by a set of explanatory variables (ie. gut origin and/or stroke). We also used methods appropriate for microbial ecology, such as Unifrac, to elucidate a phylogenetic sample distance that takes into account sequence similarity rather than just abundance. In addition to this, since Unifrac is used only at an OTU level, we also utilised Bray-Curtis similarity analysis to examine the distance between samples for the higher taxonomic levels.

**Figure 1:**
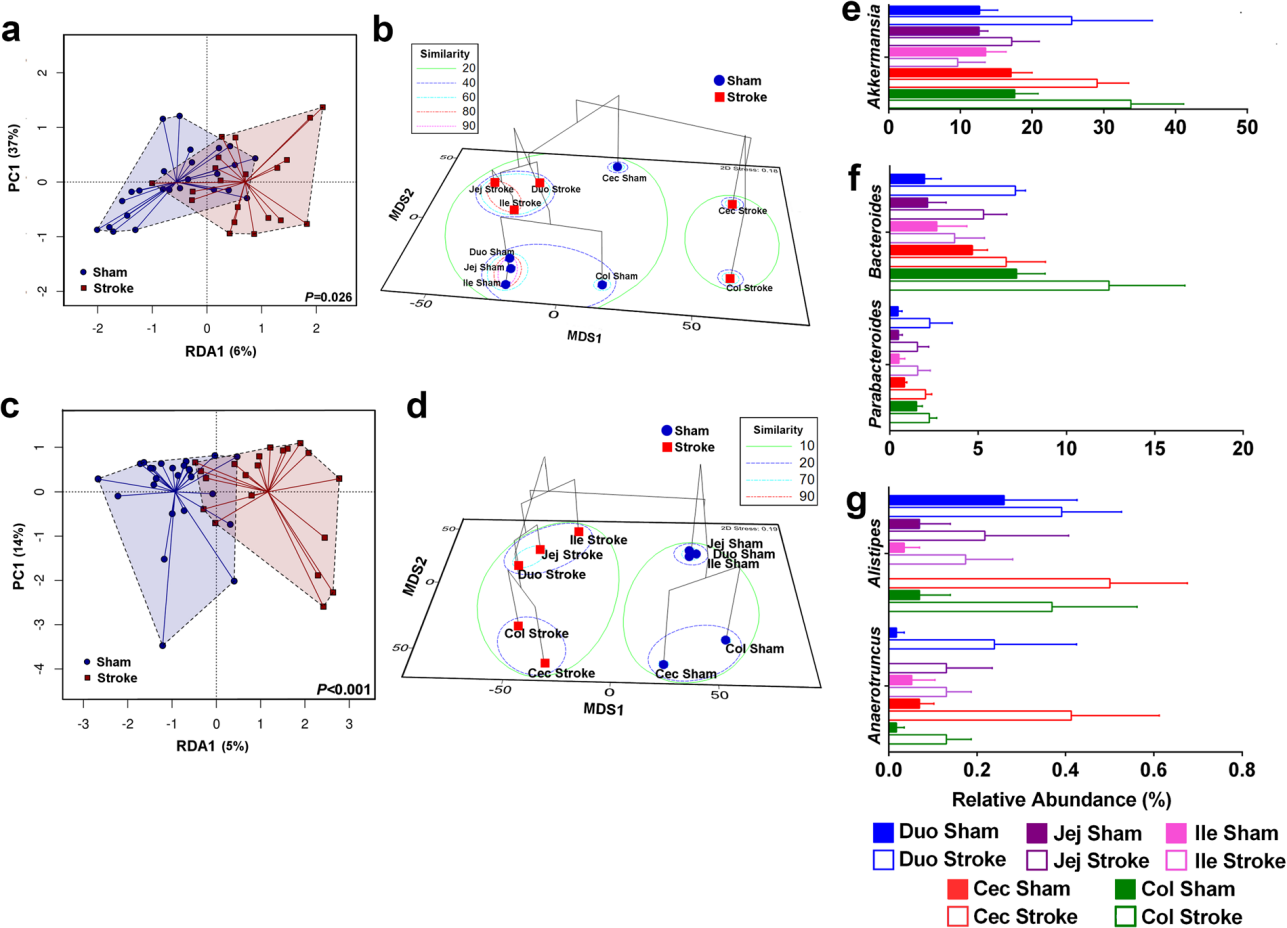
Intestinal mucosal microbiota phylum and genera are significantly disrupted in stroke. (**a**) Multivariate Redundancy Analysis RDA plot showed significant (*P* = 0.026) differences in the intestinal mucosal samples between sham-operated and post-stroke phyla. (**b**) Phylum level ANOSIM derived probabilistic pairwise group to group similarity matrix was calculated using Bray-Curtis sample to sample matrix and 99999 permutations. The ANOSIM % similarity was visualised using metric MDS. (**c**) Multivariate Redundancy Analysis RDA plot showed significant (*P* < 0.001) differences in the intestinal mucosal samples between sham-operated and post-stroke at the genus level. (**d**) ANOSIM derived probabilistic pairwise group to group similarity matrix was calculated using genus level Bray-Curtis matrix and visualised as metric MDS plot. Genera *Akkermansia* (**e**), Bacteroides, Parabacteriodes (**f**), Alistipes and Anaerotruncus (**g**) changed significantly (ANOVA) across the GIT sections after stroke. GIT sections are abbreviated in labels: duodenum (Duo), jejunum (Jej), ileum (Ile), cecum (Cec), and colon (Col). *n* = 5 per group.

Multivariate Redundancy Analysis (RDA, 999 permutations) demonstrated significant differences in the microbiota structure of the mucosa between the sham-operated and post-stroke mice at all taxonomic levels, including phylum (*P* = 0.026; Fig. 1a). However, alpha diversity indices, including Shannon and Simpson, were comparable between the experimental groups. Despite this, Multivariate two-way PERMANOVA (99999 permutations) using Bray-Curtis similarity analyses highlighted the significant differences within both factors investigated: factor 1 - GIT origin (5 levels: duodenum, jejunum, ileum, cecum, and colon; *P* = 0.043) and factor 2 - stroke (2 levels: sham-operated (control) and post-stroke, *P* = 0.025). Next, we used ANOSIM on Bray-Curtis phylum sample similarity to calculate pairwise group to group similarity on all 10 levels of data (5 origins, each for sham-operated and post-stroke). Based on multivariate ANOSIM derived probabilistic percentage similarity (Primer-E) between the groups, there was a clear separation between the duodenum, jejunum and ileum samples of sham-operated mice (similar to one another 97.6–100%) and duodenum, jejunum and ileum samples of post-stroke mice (71–85% similar; Fig. 1b). Analysis of variance at the phylum level showed an increase in Verrucomicrobia (*P* = 0.045) and decrease of Bacteroidetes (*P* = 5.3E^−3^) in the mucosal samples isolated from post-stroke mice compared to the sham-operated counterparts (Supplementary Fig. 1). The unweighted (presence/absence) abundance matrix did not differ between the groups, suggesting the presence of the same phyla across the GIT mucosal samples, but phyla abundance levels changed markedly following stroke onset.

Multivariate RDA at the genus level clearly differentiated the mucosal microbiota of sham-operated and post-stroke mice (*P* < 0.001, 999 permutations; Fig. 1c), while 2-way PERMANOVA confirmed that sham-operated and post-stroke mucosal genera are significantly different (*P* = 9E^−6^). The GIT origins could also be differentiated by 2-way PERMANOVA (*P* = 5E^−4^), and the difference due to stroke was independent of GIT origin (interaction *P* = 0.979). A graphical representation of ANOSIM pairwise group similarities showed that the mucosal samples from duodenum, jejunum and ileum of post-stroke mice were very similar to one another but clearly distinct to the sham-operated counterparts (Fig. 1d). Strikingly, ANOSIM derived probabilistic percentage similarity at the genus level between any of the sham-operated to any of the post-stroke small intestine mucosal microbiotas was lower than 10% (Fig. 1d). Analysis of variance showed significant stroke-induced elevation in the abundance of *Parabacteroides* (*P* = 7E^−4^), *Anaerotruncus* (*P* = 8.3E^−4^), *Alistipes* (*P* = 1.9E^−3^), *Akkermansia* (*P* = 0.045), and *Bacteroides* (*P* = 6.8E^−3^; Fig. 1e–g). Moreover, the most significant skew in abundance was observed in a pool of mucosal genera that could not be classified at the genus level (*P* = 2.5E^−6^, Supplementary Fig. 2), these were strongly reduced after stroke. Further analysis at an OTU level confirmed that most of the OTUs comprising these unclassified genera were closely similar to one another and belong to the order Bacteroidales.

**Figure 2:**
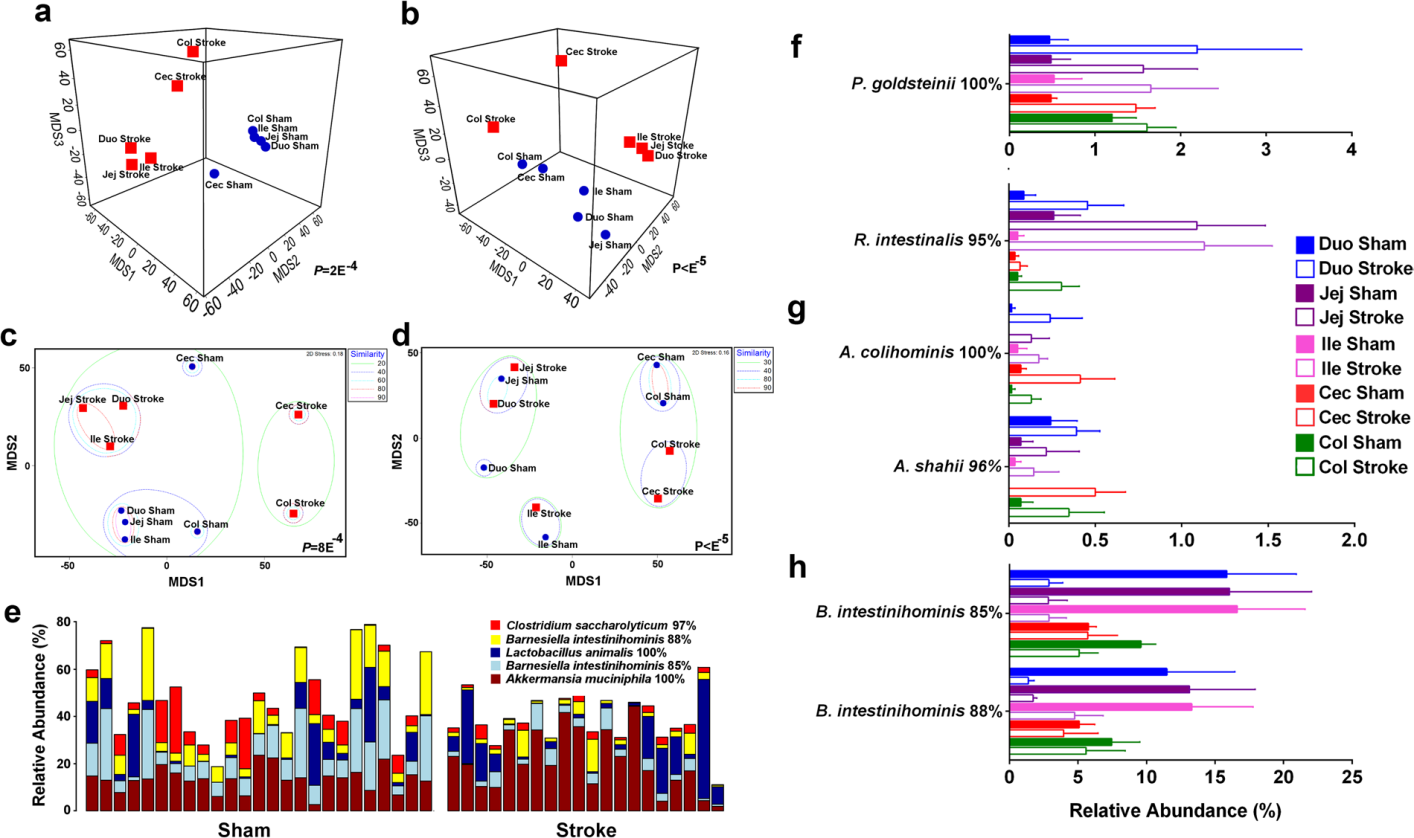
Stroke affects OTUs beta diversity and abundance. Bray-Curtis based ANOSIM pairwise group to group similarity showed significant differences after stroke on both weighted (**a**) and presence/absence (**b**) data. UniFrac confirmed significant difference between sham-operated and post-stroke samples in weighted (**c**) but not on presence/absence based unweighted UniFrac that separated samples more by origin than by stroke (**d**). (**e**) Amongst the top 5 most abundant OTUs in the intestinal microbiota, the abundance of four OTUs (with the exception of *L. animalis*) was significantly different between sham-operated and post-stroke intestinal mucosal samples (*P* < 0.038, ANOVA) Each bar represents each intestinal mucosal sample from individual mouse per group. (**f**–**h**) OTUs significantly (*P* < 0.01) affected by stroke are labelled by their closest match in NCBI 16S Microbial database and blastn % similarity across the amplified region. GIT sections are abbreviated in labels: duodenum (Duo), jejunum (Jej), ileum (Ile), cecum (Cec), and colon (Col). *n* = 5 per group.

To examine the effect of stroke on the mucosal microbiotas at the OTU level, we used weighted and unweighted UniFrac distance matrices generated in QIIME in addition to Bray-Curtis similarities. These analyses confirmed that both stroke and GIT origin significantly influenced microbiota composition in the mucosa (all 4 matrices with *P* < 0.0014). There was no significant interaction between the stroke and GIT origin in either of the analyses used. ANOSIM pairwise group-to-group similarities/distances using Bray-Curtis sample similarity on a weighted (Fig. 2a) and presence/absence matrix (Fig. 2b) separated samples by stroke rather than GIT origin. In addition, weighted UniFrac confirmed significant difference in bacterial abundance between sham-operated and post-stroke samples (Fig. 2c). However, the unweighted UniFrac, shown in Fig. 2d, demonstrated higher level of separation by the gut origin, suggesting that the presence/absence of mucosal microbiota membership was more influenced by GIT origin than whether the sample came from a sham-operated or post-stroke animal.

There were 65 OTUs significantly different (*P* < 0.05) in relative abundance between the GIT mucosal microbiotas of sham-operated and post-stroke mice; 25 of those with *P* < 0.01 (Table 1). In fact, of the 5 most prominent OTUs across the mouse GIT mucosal microbiota, 4 were differentially abundant after stroke (*P* < 0.038; Fig. 2e). Species related to *Akkermansia muciniphila* (Fig. 1e), *Parabacteroides goldsteinii* (Fig. 2f)*, Anaerotruncus colihominis, Alistipes shahii* and *Roseburia intestinalis* (Fig. 2g) demonstrated significant stroke-induced elevation in abundance. Interestingly, all of these species are relatively novel and were recently (after 2002) first described. *P. goldsteinii* is a novel anaerobe, recently characterised as pathogen identified in a few studies as a causing agent of sepsis and abdominal abscess^16^. Similarly, *A. colihominis* was first isolated and described in 2004 and implicated as pathogen 2 years later^17^. Little is known about *A. shahii* while *R. intestinalis* and *A. muciniphila* represent next generation probiotic candidates due to their heath promoting and short chain fatty acid (SCFA) producing roles. Indeed *A. muciniphila* is a potent producer of acetate^18,19^, which may be consumed by butyrate-producing *R. intestinal*is^20^. Therefore, it is possible that the two species work synergistically to promote butyrate production via metabolic cross-feeding. Increased butyrate supports epithelial heath and it is the preferred energy source for epithelial cells^21–23^. Butyrate is able to influence the epithelial expression of genes that are also stimulated by *A. muciniphila*^19^, thus enhancing each other’s stimulatory effect and mediates intestinal repair. However, intestinal repair is complex and it is unlikely that one single species is responsible. This process may be dependent on the presence of multiple beneficial species, and/or absence of pathogenic species, for optimal success. Among the phylotypes, an OTU 100% identical to *Staphylococcus sciuri* across the amplified region was present in stroke mucosa only and not in sham (*P* = 6.5E^−3^). Intriguingly, this same OTU is 100% identical to the *Staphylococcus sciuri* OTU we previously reported as 22.22 fold significantly higher in post-stroke lung compared to sham^10^.

**Table 1:** Stroke induces significant changes in OTU relative abundance in gut mucosa.

16S Microbial database hit	% ID	*P-*value	Fold higher in stroke
*Barnesiella intestinihominis*	85.00	2.50E-05	−3.38
*Barnesiella intestinihominis*	88.00	7.60E-05	−5.04
*Barnesiella intestinihominis*	88.00	0.00017	−3.09
*Clostridium indolis*	93.00	0.00018	3.67
*Barnesiella intestinihominis*	88.00	0.00021	−5.02
*Parabacteroides goldsteinii*	100.00	0.00054	2.61
*Clostridium cellulovorans*	86.00	0.00065	2.83
*Roseburia intestinalis*	95.00	7.00E-04	5.67
*Clostridium bolteae*	100.00	0.00076	16.25
*Clostridium lavalense*	96.00	0.00083	10.42
*Anaerotruncus colihominis*	100.00	0.00083	6.67
*Clostridium saccharolyticum*	95.00	0.00091	10.00
*Anaerotruncus colihominis*	95.00	0.00095	7.50
*Clostridium neopropionicum*	88.00	0.001	3.47
*Barnesiella viscericola*	84.00	0.0011	−3.94
*Clostridium indolis*	92.00	0.0015	17.50
*Clostridium indolis*	91.00	0.002	5.66
*Oscillibacter valericigenes*	94.00	0.0023	8.93
*Clostridium cellulolyticum*	87.00	0.0023	3.39
*Subdoligranulum variabile*	93.00	0.0024	3.59
*Alistipes shahii*	96.00	0.003	3.91
*Clostridium populeti*	97.00	0.0045	2.11
*Staphylococcus sciuri*	100.00	0.0065	Only in stroke
*Clostridium populeti*	97.00	0.0081	4.58
*Clostridium populeti*	97.00	0.0082	7.92
*Clostridium bolteae*	91.00	0.012	Only in sham
*Bacteroides acidifaciens*	100.00	0.012	1.96
*Oscillibacter valericigenes*	91.00	0.014	1.77
*Clostridium sporosphaeroides*	92.00	0.014	2.79
*Clostridium saccharolyticum*	93.00	0.014	−3.93
*Parabacteroides goldsteinii*	93.00	0.014	7.50
*Clostridium sporosphaeroides*	91.00	0.017	−2.90
*Clostridium saccharolyticum*	97.00	0.017	−3.52
*Clostridium saccharolyticum*	96.00	0.018	−11.20
*Akkermansia muciniphila*	100.00	0.019	−6.00
*Clostridium saccharolyticum*	93.00	0.02	−9.87
*Anaeroplasma abactoclasticum*	93.00	0.021	2.07
*Staphylococcus sciuri*	100.00	0.021	207.40
*Ruminococcus champanellensis*	89.00	0.021	3.97
*Clostridium glycolicum*	93.00	0.023	Only in stroke
*Clostridium saccharolyticum*	95.00	0.024	1.81
*Clostridium hylemonae*	98.00	0.024	−2.17
*Lactobacillus animalis*	88.00	0.026	4.38
*Clostridium saccharolyticum*	96.00	0.026	−6.53
*Ruminococcus torques*	94.00	0.028	−4.80
*Clostridium thermocellum*	89.00	0.03	−2.37
*Barnesiella intestinihominis*	85.00	0.031	−2.96
*Clostridium saccharolyticum*	96.00	0.034	Only in sham
*Clostridium aldenense*	96.00	0.034	−6.40
*Barnesiella intestinihominis*	88.00	0.035	Only in stroke
*Bacteroides acidifaciens*	99.00	0.036	1.74
*Oscillibacter valericigenes*	96.00	0.037	3.21
*Bacteroides massiliensis*	99.00	0.037	Only in stroke
*Akkermansia muciniphila*	100.00	0.038	1.56
*Ruminococcus lactaris*	97.00	0.038	3.37
*Clostridium saccharolyticum*	95.00	0.039	Only in sham
*Clostridium hathewayi*	95.00	0.04	−4.87
*Blautia luti*	95.00	0.043	Only in sham
*Ruminococcus champanellensis*	95.00	0.045	2.02
*Clostridium methylpentosum*	92.00	0.045	3.19
*Clostridium cocleatum*	97.00	0.046	Only in stroke
*Clostridium bartlettii*	95.00	0.046	Only in stroke
*Akkermansia muciniphila*	96.00	0.049	2.40
*Clostridium scindens*	93.00	0.049	Only in stroke
*Bacteroides acidifaciens*	88.00	0.049	2.68

OTUs significantly different (*P* < 0.05) in relative abundance between the GIT mucosal microbiotas of sham-operated and post-stroke mice.

Five of the OTUs significantly reduced in post-stroke mucosa had *Barnesiella intestinihominis* (85–88% sequence identity) as the closest relative within the NCBI 16S Microbial database searched using blastn (Fig. 2h). Due to the low sequence identity to the closest cultured match, the family or genus could not be assigned for these OTUs. Despite this, these OTUs aligned with 98–100% similarity to uncultured phylotypes submitted to public databases in the diet-induced mouse obesity study of Turnbaugh *et al*.^24^ and another 16S microbiota study on mouse colonic inner mucosal microbiota^25^, indicating that this group of unknown bacteria, shown to be differentially abundant in a number of studies, may play key roles in disease pathogenesis. Interestingly, obligate anaerobic bacteria belonging to the *Barnesiella* genus were shown to enable clearance of intestinal vancomycin-resistant *Enterococcus* (VRE) colonization^26^. Taken together, our findings that demonstrate significant stroke-induced reduction of OTUs resembling *Barnesiella intestinihominis* are suggestive of intestinal mucosal microbiota dysbiosis following stroke onset.

**Figure 3:**
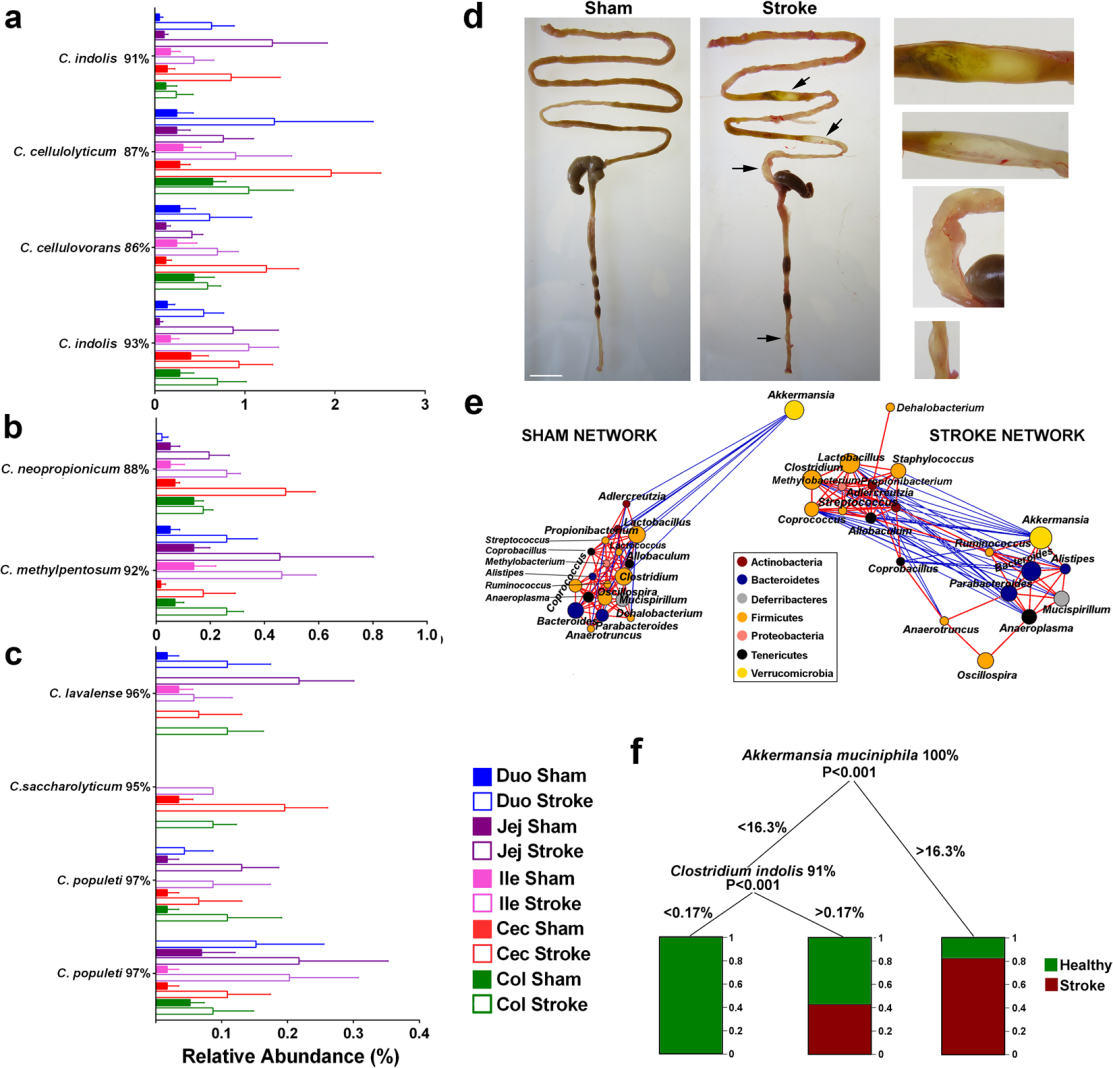
Stroke modifies clostridial species and intestinal microbial interactions. (**a**–**c**) Out of 65 OTUs influenced by stroke (*P* < 0.05), 32 had clostridia strains as their closest relatives based on blastn against 16S Microbial database. All of the significantly affected clostridia OTUs were increased in stroke in all sections of GIT mucosa. OTU IDs are replaced with the best blastn hit against 16S Microbial database and sequence similarity. (**d**) Representative image of the presence of multiple gas pockets, denoted by black arrows, in the GIT of post-stroke mice that were completely absent in sham-operated counterparts. (**e**) The network of Spearman interactions between the 20 most abundant mucosal genera in sham-operated and post-stroke GIT mucosa. Blue lines represent negative correlations and red lines represent positive correlations between genera. (**f**) One of possible Random Forest data modelling predictions on mucosal OTUs is shown. *n* = 5 per group.

Indeed, other OTUs that demonstrated significantly increased abundance (*P* < 0.01) in post-stroke mucosa were most closely related to various *Clostridium* strains by the 16S microbial database (*C. indolis, C. neopropionicum, C. cellulolyticum, C. cellulovorans, C. bolteae* and *C. populeti*), with percentage of identities across the sequenced amplicon ranging from 86 to 100% (Fig. 3a–c). The majority of OTUs and genera that were significantly different in the mucosa of sham-operated and post-stroke mice changed consistently across all of the GIT origins (Fig. 3a–c). This is in agreement with the statistical analysis of 2-way PERMANOVA, indicating that there is no difference in stroke response due to the GIT origin (insignificant interaction). The significant increase in presence of clostridia in proximity to gut barrier is in agreement with clostridial species we previously reported as migrated bacteria to post-stroke lung^10^. In fact, Table 1 of our manuscript demonstrated at 24 h after stroke onset, there is a 16-fold increase of *Clostridium bolteae* (100%ID), and this bacterium has previously been associated with gastrointestinal dysfunction in children with autism^27^. In addition, we reported a 17-fold elevation of *Clostridium indolis* (92%ID) in post-stroke mucosal microbiota. *Clostridium indolis* is a Gram-positive, motile, anaerobic, rod-shaped bacteria that has been shown to associate with infections in the intestinal tract^28^. Additionally, this dramatic increase in clostridia could explain the increased gas production observed consistently in the small intestine of post-stroke mice (Fig. 3d, Supplementary Fig. 3). It is well known that a number of exotoxin-producing clostridial species are responsible for gas gangrene^29^. Based on the amplicon sequence identity with known clostridial species (>87%), it is impossible to determine the taxonomy at a species level of these OTUs. However, it is conceivable that some of these species are novel species/strains of clostridia, which may contribute clostridial exotoxins in epithelial damage and subsequent bacterial translocation^30^.

We next used the network of Spearman-based interactions to uncover the effect of stroke on microbial interactions at the intestinal mucosa. The genus *Akkermansia* was negatively correlated with the cluster of mostly co-abundant genera in sham-operated mice (Fig. 3e). However, at 24 h after stroke, *Akkermansia* was positively correlated with *Ruminococcus*, *Alistipes, Bacteroides* and *Parabacteroides*, but negatively correlated with a number of abundant genera including *Staphylococcus and Streptococcus* (Fig. 3e). *A. muciniphila*, the first cultured intestinal bacteria from the new phylum Verrucomicrobia^18^, and recently emerged as a potential probiotic^31^. It uses mucin as a preferred source of carbon and nitrogen and produces high levels of SCFAs, acetate and propionate^18,32^, and also interacts with the host immune system^33,34^. Our finding that *A. muciniphila* was found to change its interaction profile post-stroke towards promoting beneficial (i.e. *Ruminococcus*) and suppressing pathogenic genera (i.e. *Streptococcus* and *Staphylococcus*) suggests that *A. muciniphila* may play a role in preventing pathogen migration towards the epithelial cells via active suppression, and result in reduced pathogen translocation and dissemination in the post-stroke lung^10^.

To further investigate microbial interactions following stroke onset, we performed Random Forest data modelling on mucosal OTUs. Guided by the finding that an OTU closely related to the most abundant *A. muciniphila* OTU (100% 16S rRNA gene match) was elevated in abundance in the intestinal mucosa (1.56-fold) following stroke onset (*P* = 0.038; Figs 1e and 2e), we found that almost all of the Random Forest predictions from the mucosal microbiota dataset involved *A. muciniphila*. As an example, the prediction indicated that if *A. muciniphila* was higher than 16.3% abundance, there was a greater than 80% chance that the sample originated from post-stroke mucosa (Fig. 3f).

**Figure 4:**
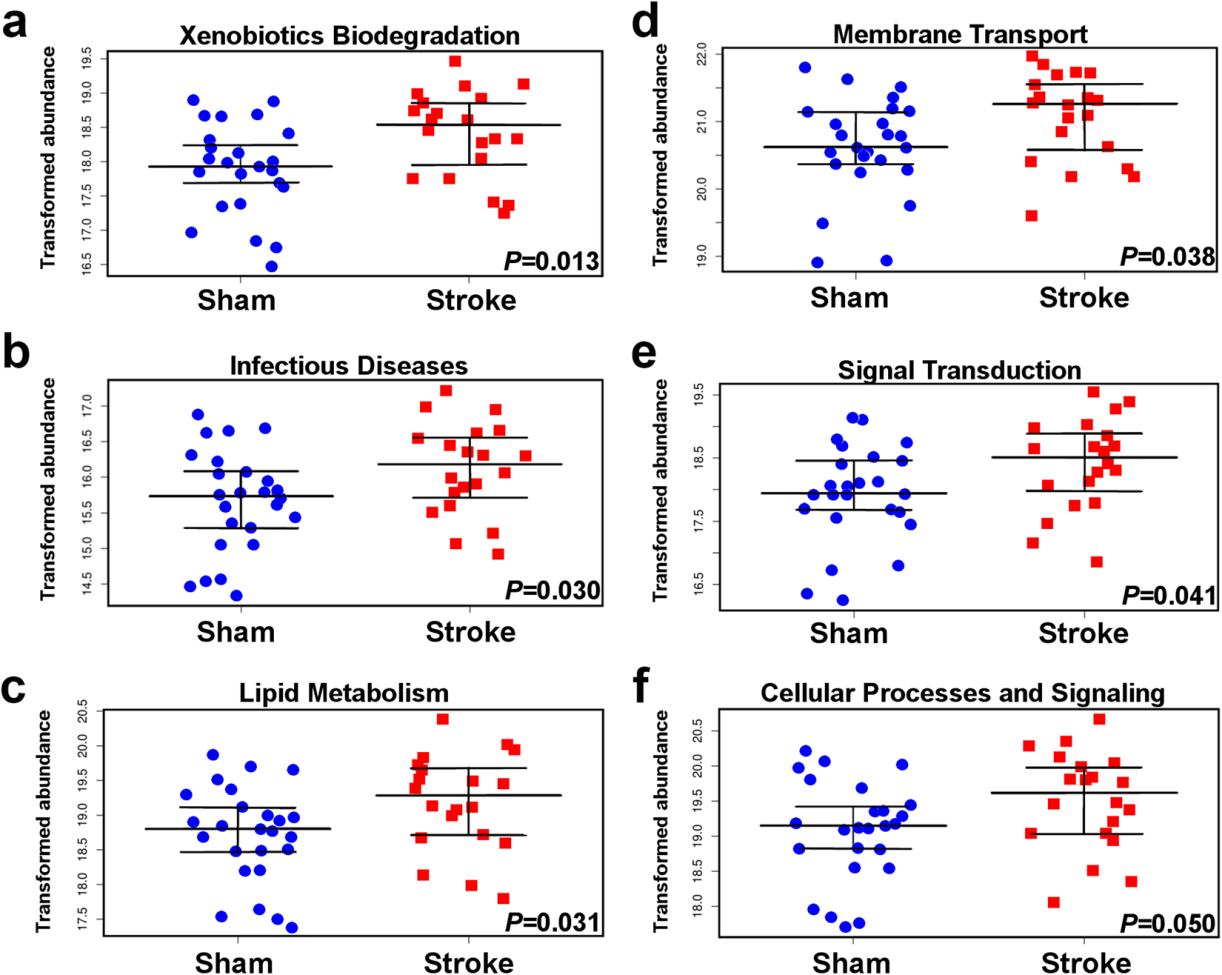
Predicted functional pathways upregulated by stroke. PICRUSt was used to analyse the differentially abundant KEGG categories (Level 2) significantly affected by stroke (*P* < 0.05), as predicted with the intestinal mucosal OTUs obtained from samples isolated from sham-operated and post-stroke mice. The analysis was performed on Galaxy server (http://huttenhower.sph.harvard.edu/galaxy) using following steps: metagenome prediction, normalisation by copy number and categorising by function. Y axes represent log2 transformed abundance. *n* = 5 per group.

Lastly, we used Phylogenetic Investigation of Communities by Reconstruction of Unobserved States (PICRUSt) to identify differentially present KEGG pathways (Level 2; *P* < 0.05) in intestinal mucosal microbiota after stroke onset. We identified 8 out of 39 KEGG predicted to be significantly upregulated at 24 h following stroke (Table 2). These included pathways associated with xenobiotics biodegradation and metabolism (Fig. 4a), infectious diseases (Fig. 4b), lipid metabolism (Fig. 4c), membrane transport (Fig. 4d), signal transduction (Fig. 4e), and cellular processes and signalling (Fig. 4f). Intriguingly, the bacterial secretion system (KEGG Level 3 pathway) from the membrane transport KEGG Level 2 category also showed stroke-mediated elevation (Supplementary Fig. 4). There was no significant differences in KEGG categories derived from individual gut origins (duodenum, jejunum, ileum, cecum, colon) between sham-operated or post-stroke mucosal microbiota. This is consistent with our 2-way PERMANOVA analysis, indicating that there is no regional specific regulation that would correlate with KEGG pathways after stroke.

**Table 2:** Predicted KEGG pathways affected by stroke-induced disruption of intestinal mucosal microbiota.

KEGG LEVEL 2 PATHWAY	*P-*VALUE
XENOBIOTICS BIODEGRADATION AND METABOLISM	0.013
INFECTIOUS DISEASES	0.03
LIPID METABOLISM	0.031
TRANSCRIPTION	0.033
MEMBRANE TRANSPORT	0.038
SIGNAL TRANSDUCTION	0.041
POORLY CHARACTERIZED	0.05
CELLULAR PROCESSES AND SIGNALING	0.05
GENETIC INFORMATION PROCESSING	0.054
CARBOHYDRATE METABOLISM	0.056
SIGNALING MOLECULES AND INTERACTION	0.057
METABOLISM	0.061
METABOLISM OF TERPENOIDS AND POLYKETIDES	0.062
NERVOUS SYSTEM	0.077
FOLDING, SORTING AND DEGRADATION	0.08
CANCERS	0.083
AMINO ACID METABOLISM	0.091
IMMUNE SYSTEM DISEASES	0.092
REPLICATION AND REPAIR	0.097
ENVIRONMENTAL ADAPTATION	0.097
METABOLISM OF OTHER AMINO ACIDS	0.1
METABOLISM OF COFACTORS AND VITAMINS	0.1
CELL GROWTH AND DEATH	0.1
TRANSLATION	0.11
NUCLEOTIDE METABOLISM	0.11
ENERGY METABOLISM	0.11
ENZYME FAMILIES	0.12
ENDOCRINE SYSTEM	0.14
NEURODEGENERATIVE DISEASES	0.15
EXCRETORY SYSTEM	0.15
CELL MOTILITY	0.16
BIOSYNTHESIS OF OTHER SECONDARY METABOLITES	0.21
GLYCAN BIOSYNTHESIS AND METABOLISM	0.22
METABOLIC DISEASES	0.24
IMMUNE SYSTEM	0.34
DIGESTIVE SYSTEM	0.35
TRANSPORT AND CATABOLISM	0.39
CIRCULATORY SYSTEM	0.57
CARDIOVASCULAR DISEASES	0.98

Phylogenetic Investigation of Communities by Reconstruction of Unobserved States (PICRUSt) was used to identify differentially present KEGG pathways (Level 2) in intestinal mucosal microbiota 24 h after stroke onset.

It is noteworthy that one of the OTUs (with 96% identical to *A. muciniphila*) significantly increased in the post-stroke intestinal mucosa (*P* = 0.049, 2.4 fold) was the same OTU previously reported to be elevated by 7.8 fold in the lungs of post-stroke mice^10^. The similarity of the changes we observed in small intestine mucosal microbiota to those reported in post-stroke lung tissue is striking and suggests the notion that the small intestine mucosa is a battleground and a hotspot for the bacterial translocation into the lung after stroke. In fact, we have previously performed principal coordinate analysis on pairwise ANOSIM similarities of various tissues in sham-operated and post-stroke mice^10^. The ANOSIM analysis was based on Weighted UniFrac matrix, and we can clearly showed that the microbial communities in the lung and tongue are closely associated in sham-operated mice, however this association was lost at 24 h following stroke onset. To examine whether the microbial communities in the lung are derived from coprophagic activities, we have previously shown that post-stroke mice had a lower capacity to aspirate, and they demonstrated similar levels of translocation of aspired contents to the systemic circulation when compared to their sham-operated counterparts^10^. Despite this, it should be noted that a previous landmark study found that aspiration of only 200 CFUs of *S. pneumoniae* was sufficient to induce pneumonia and bacteraemia in post-stroke mice, compared to 200,000 CFUs needed in sham-operated mice^35^. Taken together, although our findings support the view that the microbial communities in the lung are not derived from coprophagic activities, it is however feasible that small amounts of bacteria reaching the lower airways in post-stroke mice may also lead to pneumonia and changes in the microbiota.

There is no doubt about the role of known lung pathogens in post-stroke pneumonia, the role of increased *A. muciniphila*, as well as *Lactobacillus* (Fig. 2e)^10^, found in post-stroke intestinal mucosa and lung is unclear. Although numerous *Lactobacillus* have been reported to cause opportunistic infections^36^, the elevation of *Akkermansia* in the post-stroke intestinal mucosa and lung may support *Akkermansia*-assisted healing of wound damage^13,14,37^, strengthen epithelial integrity^37^ and control translocation. Therefore, further work to investigate the possible role of *Akkermansia* in post-stroke tissue repair may reveal novel functions. With the exponential growth of research and interest in gut microbiota, their roles are likely to be soon recognized. The levels of communication and interactions between the beneficial bacteria and the host epithelial and immune cells is a fascinating topic for further research. Additionally, functional predictions also indicate that bacterial interactions with the host also play significant role in the post-stroke pneumonia onset via gut bacterial translocation control, further strengthening the need to investigate the basis of stroke-mediated intestinal molecular processes.

There are recent reports demonstrating changes in the composition of faecal microbiota after stroke^15,38,39^. However, the mucosal bacterial cohort is very distinct in both role and membership from the luminal and faecal microbiota, and mucosa-associated bacteria are often overlooked. Therefore, in this study, we provide a detailed insight into the composition of the mucosal microbiota after stroke. Being closest to the host epithelium, we propose that the mucosal microbiota is the prime location to respond to stroke injury and potentially has the opportunity to either invade the host or launch epithelial healing responses to re-establish gut barrier integrity after stroke.

## Conclusions

We revealed the mucosa of the gastrointestinal tract is significantly and robustly modified following the onset of stroke. The main characteristics of the stroke-induced shift in composition of mucosal microbiota were an increased abundance of clostridial species and elevation of *Akkermansia muciniphila*. Furthermore, the alterations in mucosal microbiota composition following stroke were also found to change the predicted functional potential of the microbiota, with significant increases in pathways associated with infectious diseases, membrane transport, xenobiotic degradation, lipid metabolism and signalling. A better understanding of the precise molecular events leading to stroke-induced mucosal microbiota alterations may identify novel therapeutic targets to improve patient outcomes.

## Methods

### Mice

Males of 7–10 weeks old C57BL/6J mice were obtained from Monash Animal Services and housed under specified pathogen-free (SPF) conditions in Monash University. Following transportation, mice were acclimatized for a minimum period of 7 days before use. All mice were housed in groups of no more than 5 after weaning in a 12-hour light-dark cycle in a temperature controlled environment. Water and food pellets (Irradiated Rat and Mouse, Specialty Feeds, Australia) were provided *ad libitum* and their cages were changed weekly. All animal experiments were approved by the Monash University Animal Ethics Committee (MMCB/2014/29 and MMCB/2014/30), and that all methods were performed in accordance with the relevant guidelines and regulations.

### Mouse focal cerebral ischemia model

Mice underwent the mid-cerebral artery occlusion (MCAO) model of cerebral ischemia-reperfusion injury (stroke) as previously described^40^. Briefly, the mice were anesthetized by intraperitoneal injection of a mixture of 10 mg/kg xylazine hydrochloride (Lyppard Australia) and 200 mg/kg ketamine hydrochloride (Lyppard Australia). Body temperature was maintained at 37 °C using a heating pad and temperature regulator with rectal probe. Mice were randomly divided into 2 groups, stroke-operated and sham-operated. All surgical instruments were sterilized before the surgery. Before any incision, the area was swabbed with ethanol. To induce stroke, a 10 mm incision was made on the right side of the mouse and the common carotid artery, external carotid artery, and internal carotid artery were dissected free. The external carotid artery was further dissected distally, then coagulated and cut to serve as a stump. After applying temporary clamps at the common carotid artery and internal carotid artery, a monofilament with a silicon coating, diameter of 0.21–0.23 mm, was inserted into the stump of the external carotid artery. The monofilament was then advanced a defined distance (12 mm) so that its distal end came to rest across the origin of the MCA. At this stage, a laser Doppler perfusion monitor (Perimed) on the cranium of each mouse was used to verify occlusion of the MCA. A drop in the pre-occlusion perfusion reading of more than 70% was considered a successful occlusion and included in the study. The stump of the external carotid artery was tied off. The wound on the neck of the mouse was sutured and the mouse was then transferred onto a heat pad to maintain its body temperature at 37 °C. After 60 mins of occlusion, the monofilament was withdrawn to allow reperfusion to occur. The animal then recovered from the anaesthesia. Dishes of mash and water, as well as food pellets were placed in the cage after surgery as the mice recovered and access to water was provided. Sham-operated animals were subjected to the initial anaesthetic and neck incision only. All animals were put onto a 37 °C heat pad post-surgery to recover from anaesthesia. All animals included in the study were randomly assigned to go into sham-operated or stroke-operated groups, researchers were not blinded to sample identification. No animals were excluded. All mice were housed individually in clean cages after surgery until culled. There was approximately 10% mortality rate in the animals of stroke-operated group, while all animals survived the sham surgery.

### Microbiota and statistical analyses

At 24 h after reperfusion, sham-operated and stroke-operated mice were culled and the duodenum, jejunum, ileum, cecum and colon removed, cleaned with sterile PBS and mucosal samples obtained by scaping with a sterile blade. The samples were immediately frozen in liquid nitrogen and stored at −80 °C until DNA isolation was performed. Total DNA was isolated using Bioline ISOLATE II Genomic DNA Kit (#BIO-52067) according to the Bioline protocol. PCR (30 cycles), using 50 ng of tissue derived DNA as template, was performed using Q5 DNA polymerase (New England Biolabs) with a primer set selected to amplify V3-V4 region of 16S rRNA gene (forward: ACTCCTACGGGAGGCAGCAG and reverse: GGACTACHVGGGTWTCTAAT). Equal quantities of each amplicon were pooled and sequencing was performed on an Illumina MiSeq (2 × 300 bp), following the method detailed by Fadrosh *et al*.^41^.

Analysis of microbial communities was completed using QIIME v.1.9.1^42^ with the analysis parameters detailed in Stanley *et al*.^10^, and following the analysis pipeline described previously by Jervis-Bardy *et al*.^43^. Taxonomies for the mucosal microbiota dataset were assigned using the GreenGenes database^44^. Further analysis was performed using QIIME, Primer-E, R Party package, PICRUSt^45^, blastn against 16S Microbial NCBI database and Calypso^46^. Random Forest predictions were done using the R package “party” and cforest function on 44 observations, 655 variables (mucosal OTUs) and 500 random forest trees per prediction. The final OTU table was square root transformed and Total Sum Scaling (TSS) normalised for all of the statistical analysis.

### Sequencing inclusion and exclusion criteria

OTUs with relative abundance of less than 0.01% and samples with less than 1150 sequences were removed from the analysis. Although the experiment was completed with n = 5 per group, removal of samples with low sequence number resulted in a total of 25 and 20 successfully sequenced gut region samples for sham-operated and post-stroke mice, respectively. Specifically, the duodenum mucosal sample of post-stroke mouse #5; the jejunum mucosal sample of post-stroke mouse #4; the ileal mucosal sample of post-stroke mouse #4; the cecal mucosal sample of post-stroke mouse #3; the colonic mucosal sample of post-stroke mouse #4 were removed from analysis. Therefore, the excluded samples were not from the same mouse or intestinal region.

### Sequencing data availability

All of the sequencing data are publically available in the Metagenomics Analysis Server (MG-RAST) database under project ID mgp16019 and metagenome ID mgm4707925.3.

### Data availability

All of the sequencing data are submitted to the Metagenomics Analysis Server (MG-RAST) under project titled “Evidence for translocation and dissemination of commensal bacteria as a source of infection after stroke”, which consists of 2 datasets (4675190.3 and 4707925.3).

### Ethics approval and consent to participate

All animal experiments were approved by the Monash University Animal Ethics Committee (MMCB/2014/29 and MMCB/2014/30).

## Electronic supplementary material

Supplemental material

## References

[1] Chamorro A, Urra X, Planas AM (2007). Infection after acute ischemic stroke: a manifestation of brain-induced immunodepression. Stroke.

[2] Meisel C, Schwab JM, Prass K, Meisel A, Dirnagl U (2005). Central nervous system injury-induced immune deficiency syndrome. Nature reviews. Neuroscience.

[3] Marik PE (2001). Aspiration pneumonitis and aspiration pneumonia. The New England journal of medicine.

[4] Kalra L (2015). Prophylactic antibiotics after acute stroke for reducing pneumonia in patients with dysphagia (STROKE-INF): a prospective, cluster-randomised, open-label, masked endpoint, controlled clinical trial. Lancet.

[5] Westendorp WF (2015). The Preventive Antibiotics in Stroke Study (PASS): a pragmatic randomised open-label masked endpoint clinical trial. Lancet.

[6] Tziomalos K (2016). Prophylactic antibiotic treatment in severe acute ischemic stroke: the Antimicrobial chemopRrophylaxis for IschemicSTrokE In MaceDonIa-Thrace Study (ARISTEIDIS). Internal and emergency medicine.

[7] Shim R, Wong CH (2016). Ischemia, Immunosuppression and Infection–Tackling the Predicaments of Post-Stroke Complications. International journal of molecular sciences.

[8] Winek K (2016). Depletion of Cultivatable Gut Microbiota by Broad-Spectrum Antibiotic Pretreatment Worsens Outcome After Murine Stroke. Stroke.

[9] Benakis C (2016). Commensal microbiota affects ischemic stroke outcome by regulating intestinal gammadelta T cells. Nat Med.

[10] Stanley D (2016). Translocation and dissemination of commensal bacteria in post-stroke infection. Nat Med.

[11] Ouwerkerk JP, de Vos WM, Belzer C (2013). Glycobiome: bacteria and mucus at the epithelial interface. Best Pract Res Clin Gastroenterol.

[12] Dongarra ML (2013). Mucosal immunology and probiotics. Curr Allergy Asthma Rep.

[13] Alam A (2016). O-012 The Intestinal Wound Regeneration Modulates Mucosal Microenvironment to Stimulate Expansion of a Local Pro-restitutive Microbiota. Inflamm Bowel Dis.

[14] Alam A (2016). The microenvironment of injured murine gut elicits a local pro-restitutive microbiota. Nature microbiology.

[15] Singh V (2016). Microbiota Dysbiosis Controls the Neuroinflammatory Response after Stroke. The Journal of neuroscience: the official journal of the Society for Neuroscience.

[16] Awadel-Kariem FM, Patel P, Kapoor J, Brazier JS, Goldstein EJ (2010). First report of *Parabacteroides goldsteinii* bacteraemia in a patient with complicated intra-abdominal infection. Anaerobe.

[17] Lau SK (2006). Bacteraemia caused by *Anaerotruncus colihominis* and emended description of the species. J Clin Pathol.

[18] Derrien M, Vaughan EE, Plugge CM, de Vos WM (2004). *Akkermansia muciniphila* gen. nov., sp. nov., a human intestinal mucin-degrading bacterium. Int J Syst Evol Microbiol.

[19] Lukovac S (2014). Differential modulation by *Akkermansia muciniphila* and Faecalibacterium prausnitzii of host peripheral lipid metabolism and histone acetylation in mouse gut organoids. mBio.

[20] Duncan SH, Hold GL, Barcenilla A, Stewart CS, Flint HJ (2002). *Roseburia intestinalis* sp. nov., a novel saccharolytic, butyrate-producing bacterium from human faeces. Int J Syst Evol Microbiol.

[21] Brahe LK, Astrup A, Larsen LH (2013). Is butyrate the link between diet, intestinal microbiota and obesity-related metabolic diseases?. Obesity reviews: an official journal of the International Association for the Study of Obesity.

[22] Meijer K, de Vos P, Priebe MG (2010). Butyrate and other short-chain fatty acids as modulators of immunity: what relevance for health?. Curr Opin Clin Nutr Metab Care.

[23] Louis P, Flint HJ (2009). Diversity, metabolism and microbial ecology of butyrate-producing bacteria from the human large intestine. FEMS Microbiol Lett.

[24] Turnbaugh PJ, Backhed F, Fulton L, Gordon JI (2008). Diet-induced obesity is linked to marked but reversible alterations in the mouse distal gut microbiome. Cell Host Microbe.

[25] Musch MW, Wang Y, Claud EC, Chang EB (2013). Lubiprostone decreases mouse colonic inner mucus layer thickness and alters intestinal microbiota. Dig Dis Sci.

[26] Ubeda C (2013). Intestinal microbiota containing Barnesiella species cures vancomycin-resistant *Enterococcus faecium* colonization. Infect Immun.

[27] Pequegnat B (2013). A vaccine and diagnostic target for *Clostridium bolteae*, an autism-associated bacterium. Vaccine.

[28] Biddle AS (2014). The complete genome sequence of *Clostridium indolis* DSM 755(T.). Standards in genomic sciences.

[29] Stevens DL, Aldape MJ, Bryant AE (2012). Life-threatening clostridial infections. Anaerobe.

[30] Keyburn AL (2008). NetB, a new toxin that is associated with avian necrotic enteritis caused by *Clostridium perfringens*. PLoS Pathog.

[31] Cani PD, Everard A (2014). *Akkermansia muciniphila*: a novel target controlling obesity, type 2 diabetes and inflammation?. Med Sci (Paris).

[32] Derrien M, Collado MC, Ben-Amor K, Salminen S, de Vos WM (2008). The Mucin degrader *Akkermansia muciniphila* is an abundant resident of the human intestinal tract. Appl Environ Microbiol.

[33] Derrien M, Belzer C, de Vos WM (2017). *Akkermansia muciniphila* and its role in regulating host functions. Microbial pathogenesis.

[34] Derrien M (2011). Modulation of Mucosal Immune Response, Tolerance, and Proliferation in Mice Colonized by the Mucin-Degrader *Akkermansia muciniphila*. Frontiers in microbiology.

[35] Prass K, Braun JS, Dirnagl U, Meisel C, Meisel A (2006). Stroke propagates bacterial aspiration to pneumonia in a model of cerebral ischemia. Stroke.

[36] Martinez RM, Hulten KG, Bui U, Clarridge JE (2014). Molecular analysis and clinical significance of Lactobacillus spp. recovered from clinical specimens presumptively associated with disease. Journal of clinical microbiology.

[37] Reunanen J (2015). *Akkermansia muciniphila* Adheres to Enterocytes and Strengthens the Integrity of the Epithelial Cell Layer. Appl Environ Microbiol.

[38] Houlden A (2016). Brain injury induces specific changes in the caecal microbiota of mice via altered autonomic activity and mucoprotein production. Brain, behavior, and immunity.

[39] Yamashiro K (2017). Gut dysbiosis is associated with metabolism and systemic inflammation in patients with ischemic stroke. PLoS One.

[40] Wong CH, Jenne CN, Lee WY, Leger C, Kubes P (2011). Functional innervation of hepatic iNKT cells is immunosuppressive following stroke. Science.

[41] Fadrosh DW (2014). An improved dual-indexing approach for multiplexed 16S rRNA gene sequencing on the Illumina MiSeq platform. Microbiome.

[42] Caporaso JG (2010). QIIME allows analysis of high-throughput community sequencing data. Nature methods.

[43] Jervis-Bardy J (2015). Deriving accurate microbiota profiles from human samples with low bacterial content through post-sequencing processing of Illumina MiSeq data. Microbiome.

[44] DeSantis TZ (2006). Greengenes, a chimera-checked 16S rRNA gene database and workbench compatible with ARB. Applied and environmental microbiology.

[45] Langille MG (2013). Predictive functional profiling of microbial communities using 16S rRNA marker gene sequences. Nat Biotechnol.

[46] Zakrzewski M (2017). Calypso: a user-friendly web-server for mining and visualizing microbiome-environment interactions. Bioinformatics.

