# Supplementary material for: An insight into intestinal mucosal microbiota disruption after stroke
Source: Sci Rep. 2018 Jan 12;8:568. doi: 10.1038/s41598-017-18904-8 (PMC5766598; doi:10.1038/s41598-017-18904-8)
Supplement: Supplementary file 1 — Supplemental material [file 41598_2017_18904_MOESM1_ESM.pdf]

An insight into intestinal mucosal microbiota disruption after stroke

Dragana Stanley[1], Robert J. Moore[2,3] and Connie H. Y. Wong[4,§]

1. School of Health Medical and Applied Sciences, Central Queensland University, Bruce Highway, Rockhampton, Queensland 4702, Australia. Email: d.stanley@cqu.edu.au
2. School of Science, RMIT University, Bundoora, Victoria 3083, Australia. Email: rob.moore@rmit.edu.au
3. Infection and Immunity Program, Biomedicine Discovery Institute, Department of Microbiology, Monash University, Clayton, Victoria 3800, Australia
4. Centre for Inflammatory Diseases, Department of Medicine, School of Clinical Sciences, Monash University, Clayton, Victoria 3168, Australia. Email: connie.wong@monash.edu

[§]Address for correspondence: Connie H. Y. Wong, PhD., Centre for Inflammatory Diseases, Department of Medicine, School of Clinical Sciences, Monash Medical Centre, Monash University, Clayton, VIC 3168 Australia; Email: connie.wong@monash.edu

SUPPLEMENTARY FIGURES: 4

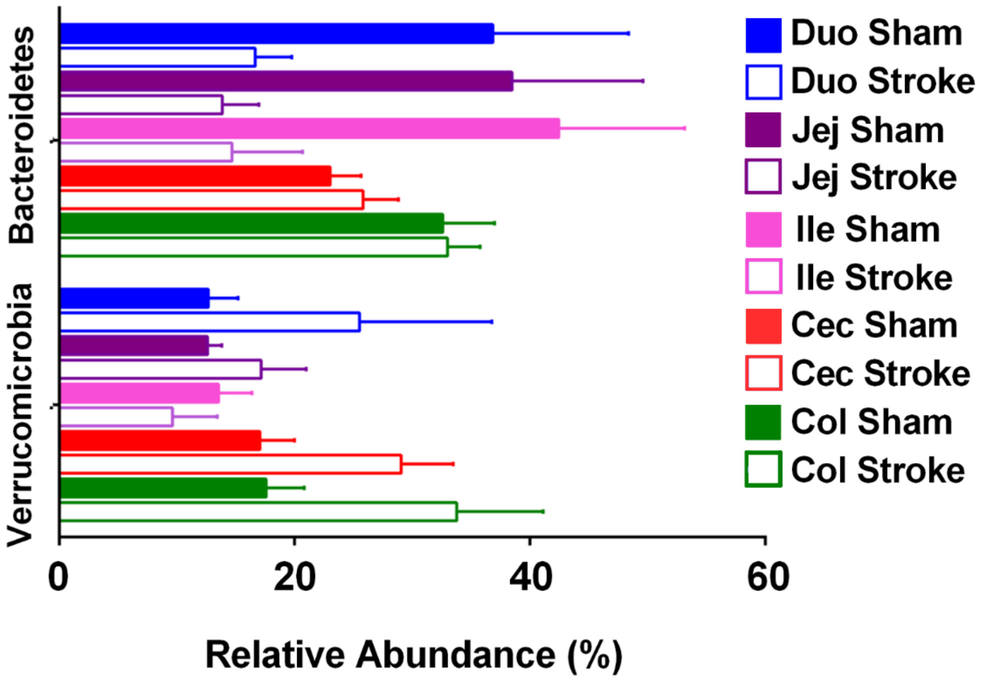

Supplementary Figure 1: Phylum Bacteroidetes was significantly decreased (P=5.3E^{-3}) and Verrucomicrobia (P=0.045) were markedly increased in the intestinal mucosal samples isolated from post-stroke mice as compared to sham-operated counterparts, consistently across all 5 GIT sections. n = 5 per group.

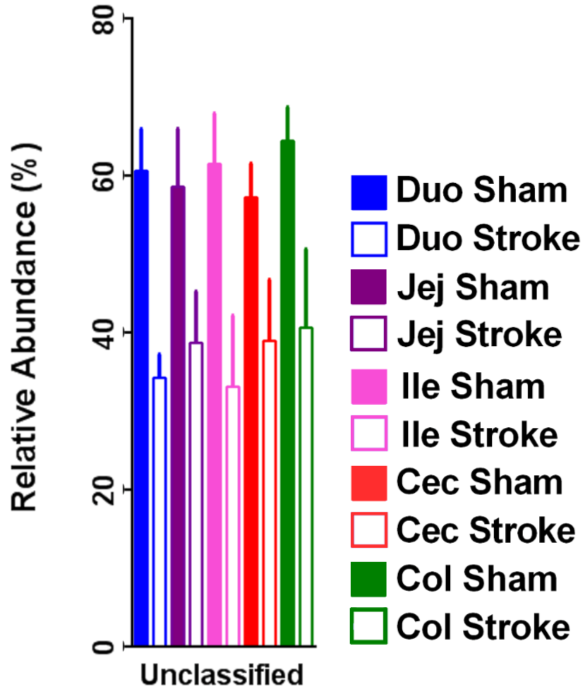

Supplementary Figure 2: Genera Unclassified elevated significantly (ANOVA) across the GIT sections after stroke. GIT sections are abbreviated in labels: duodenum (Duo), jejunum (Jej), ileum (Ile), cecum (Cec), and colon (Col). n = 5 per group.

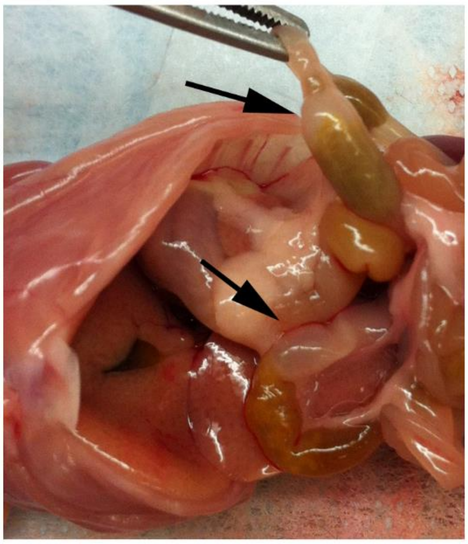

Supplementary Figure 3: Representative image of the presence of multiple gas pockets, denoted by black arrows, in the small intestine of post-stroke mice that were completely absent in sham-operated counterparts.

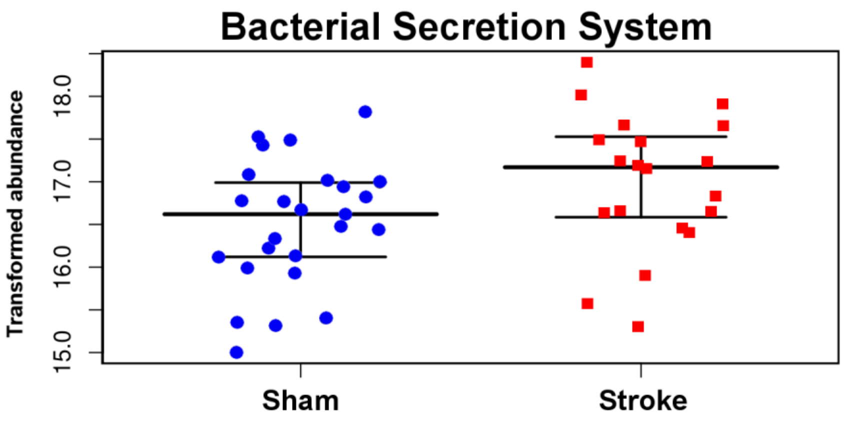

Supplementary Figure 4: KEGG level 3 *Bacterial Secretion System* category was significantly (*P*=0.042) higher represented in stroke than in sham mucosa. Y axes represents log2 transformed functional category abundance.
